# Primary gastric synovial sarcoma: A case report and literature review

**DOI:** 10.1016/j.ijscr.2020.12.055

**Published:** 2020-12-26

**Authors:** Charles Marchand Crety, Sara Bellefqih, Koceila Amroun, Christian Garbar, Felix Felici

**Affiliations:** a Department of Radiotherapy, Institut de Cancérologie Jean Godinot, 1 Rue du General Koenig, Insitut Godinot, 51100, Reims, France; b Department of Surgery, Institut de Cancérologie Jean Godinot, 1 Rue du General Koenig, Insitut Godinot, 51100, Reims, France; c Department of Pathology, Institut de Cancérologie Jean Godinot, 1 Rue du General Koenig, Insitut Godinot, 51100, Reims, France

**Keywords:** SS, synovial sarcoma, g/dl, gram per deciliter, FNCLCC, French Federation of Cancer Centers Sarcoma Group, PCR, *Polymerase Chain Reaction*, DNA, desoxyribo- nucleic acid, RNA, ribonucleic acid, GIST, gastrointestinal stromal tumour, c-Kit, mast/stem cell growth factor receptor, FISH, fluorescence in situ hybridization, Synovial sarcoma, Gastric synovial sarcoma, Digestive surgery, Molecular pathological diagnosis

## Abstract

**Introduction:**

Synovial sarcoma (SS) is a mesenchymal neoplasm that is characterized by its unique histological pattern and is most commonly found near the joints of the extremity. Stomach involvement is very rare. This work aimed to present the case of a patient with gastric SS. We also conducted a review of 39 gastric SS cases reported in the literature.

**Presentation of case:**

Here we report a case of primary gastric synovial sarcoma in a 32-year-old male patient revealed by gastric reflux. Partial gastrectomy was performed showing a 35 mm lesion with a high spindle cell component. Immunohistochemistry revealed 18q11.2 translocation expression in most of the cells asserting a diagnosis of SS. No local or distant recurrence occurred at 8 months post-operative follow-up.

**Discussion:**

The majority of SS occurs in the extremities and is most often associated with tendons in the large articulations of young adults. Gastric SS are very scarce and a molecular biology approach to detect the SYT-SSX fusion gene is required for conclusive diagnosis. We carried out a clinical review of the 40 cases of primary gastric SS, including our case. They all underwent an excisional surgery, most of them by partial gastectomy or wedge resection. Recurrences were rare and early when they occurred.

**Conclusion:**

Gastric SS is a very uncommon neoplasia although it is henceforth a described entity. Immunohistochemical detection of a pathognomonic translocation is needed to make the diagnosis of SS. Best therapeutic approach for these tumors remains surgical resection with no specific excisional technique recommended.

## Highlights

• Primary synovial sarcoma (SS) of the stomach is a very rare disease.
• To date, only 39 gastric SS cases have been reported in the literature.
• Here is a report of a surgically resected primary gastric SS case and a review of the corresponding literature.

## 1 Introduction

Synovial sarcoma (SS) is a malignant mesenchymal neoplasm representing less than 10% of all soft tissue sarcomas. It usually occurs in the second and third decade of life [1]. A pathognomonic chromosomal translocation generating SYT-SSX fusion transcripts genetically characterize SS and the detection of this translocation leads to the diagnosis of SS [2]. Although not related to the synovium, it is located near the joints of the limbs in 80–90% of the cases and rarely, the digestive tract [3]. Primary SS of the stomach is a very rare disease. To date, only 39 gastric SS cases have been reported in the literature to our knowledge. The following is a report of a primary gastric SS case and a review of the corresponding literature. The report has been arranged in line with SCARE guidelines [4].

## 2 Presentation of case

**Fig. 1:**
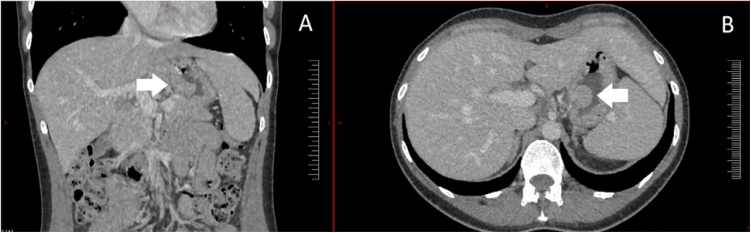
Coronal (A) and axial (B) CT-scan images showing a 25 mm long axis lesion located on the small curvature of the stomach under the cardia.

**Fig. 2:**
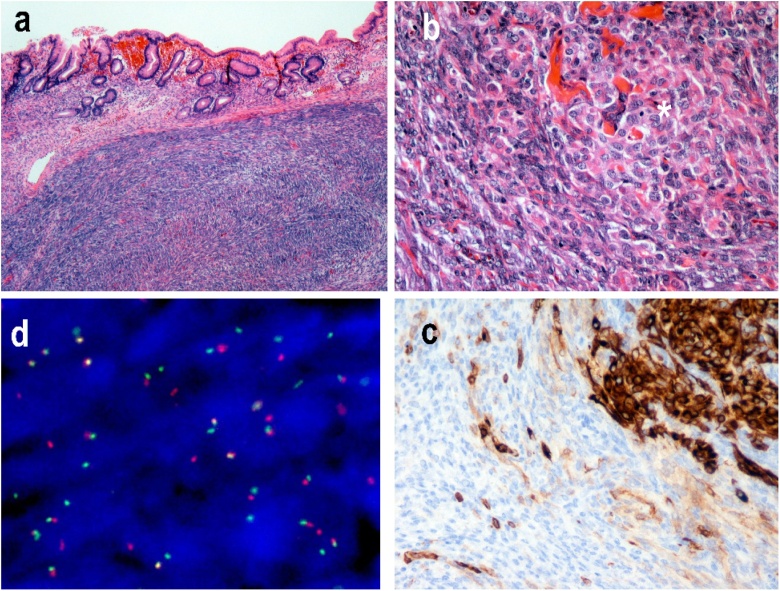
a) Submucosal infiltration of atypical spindle cells (hematoxylin eosin staining; 4X magnification). b) Epithelial neoplastic cell elements (star; hematoxylin eosin staining; 20x magnification). c) Cytokeratin-positive epithelial cell elements (cytokeratin AE1/AE3 (Agilent/Dako®) Immunohistochemistry; 20x magnification). d) Specific translocation 18q11.2 with red and green break probes (SS18 (Zytovision®), Fluorescence in situ hybridization).

We present the case of a 32 years-old female patient with no personal or family medical history, who was admitted for the first time for gastric reflux causing chest and interscapular pain, associated with normocytic anemia (hemoglobin level was 8.7 g/dl), referred by his general practitioner. The pain was relieved by the use of proton-pump inhibitors. A one-time melena episode also occurred in the history of the disease. She underwent esogastric endoscopy, revealing a 1.5 cm diameter submucosal polyp with central dip, localized under the cardia. Echoendoscopy showed a hypoechogenic submucous lesion with central ulceration, reminding a neurilemmoma in the first place. A thoraco-abdomino-pelvic CT revealed this 25 mm gastric tumor, with low portal phase contrast enhancement (Fig. 1) without lymph node involvement or remote lesions. Biopsies were performed during both endoscopy exams, and pathological examination indicated FNCLCC grade 2 spindle cells sarcoma, with non-specific morphology and phenotype. The tumorous cells were negative for actin, caldesmone, CD34, CD117, DOG1 and PS100. The patient is then referred to a regional Cancer Centre and partial gastrectomy was performed by a an experienced digestive surgeon specialized in cancer surgery, showing a 35 × 25 × 13 mm lesion with a high spindle cell component (Fig. 2). Immunohistochemistry (FISH test) revealed 18q11.2 translocation expression in 90% of the cells asserting a diagnosis of SS. Surgical margin was negative. Anatomopathological examination of the surgical specimen is illustrated in Fig. 2. The day after the operation, the patient complained of abdominal pain; an X-ray of the abdomen showed pneumoperitoneum. The patient therefore underwent needle exsufflation to drain this post-operative pneumoperitoneum. The pain subsided the next day and the patient was discharged from hospital five days after the operation. A preventive anticoagulation with low molecular weight heparin (4000 anti-Xa IU per day) was prescribed for the month following the surgery as well as a blood test at 1 month and 3 months. Follow-up visits with physical examination were scheduled at 1 month after surgery and then every 3 months in the regional Cancer Center where the patient was operated on. Four months after the surgery, initial gastric reflux causing chest and interscapular pain completely disappeared and hemoglobin level normalized to 12.3 g/dl. No adjuvant treatment was given. There was no recurrence at 8 months of follow-up.

## 3 Discussion

The majority of SS occurs in the extremities (80%) and is most often associated with tendons in the large articulations of young adults. The term "synovial" originally comes from the frequency of association of the tumor with the major joints of the extremities and with a histological appearance resembling the development of the synovium. Yet, this association has been questioned by immunohistochemical examinations. Currently, although a study on transgenic mice indicated that SS could be derived from immature myoblast, the tissues of origin remain unclear [5]. Other locations have also been identified, including the lung, heart, kidneys, prostate, mediastinum and peritoneum. SS are also identified in association with the gastrointestinal tract, including the esophagus and small intestine [1,6,7].

A molecular biology approach to detect the SYT-SSX fusion gene is required for conclusive diagnosis. The PCR method allows the identification of this fusion gene or sequence from DNA or RNA from tumor tissues in the most cases [8]. GISTs need to be distinct from SS. The vast proportion of GISTs is identified on the expression of the c-Kit. Synovial sarcomas are c-Kit negative on immunohistochemical analysis as shown in several studies. Weak or negative lesions for c-Kit should seek expression of a SYT–SSX fusion protein.

To the best of our knowledge, only 39 cases of primary gastric SS are reported in the literature. Only one case of metastatic gastric involvement secondary to SS has been described [[9], [10], [11], [12], [13], [14], [15], [16], [17]]. A clinical review of the 40 cases of primary gastric SS, including our case, is shown in Table 1. Among these cases, median age was 44 (range 13–68). The male-female ratio was 1.1 and the median size of the primary SS was 43 mm (range 8–160). Epigastric pain and anemia are the most common clinical presentations of gastric synovial sarcomas. The vast majority of these SS cases were monophasic subtypes (86%). Three cases (10%) underwent total gastrectomy, eleven (35%) partial gastrectomy, nine (29%) wedge resection and eight (26%) tumor resection. Only 7 patients (24%) received adjuvant chemotherapy. Recurrences were rare and early (<18 months after surgery) when they occurred. This is consistent with the fact that the majority of recurrences of soft tissue sarcomas occur within 3 years after treatment [18].

**Table 1:** Clinical feature and outcome of 40 reported cases of primary gastric synovial sarcoma.

No. of Cases	Year, First author	Age	Sex	Size (mm)	Subtype	Type of surgery	Adjuvant treatment	Outcome
1	2000, Billings	47	M	52	Biphasic	Partial Gastrectomy	No	AWOD at 1.8-y
2	2000, Billings	55	F	160	Monophasic	Partial gastrectomy	No	DFD (6-m)
3	2007, Akhunji	42	M	115	Biphasic	Tumor resection	Chemotherapy	DFD (2-y)
4	2008, Makhlouf	67	F	8	Monophasic	Partial gastrectomy	No	AWOD at 1-y
5	2008, Makhlouf	49	M	20	Monophasic	Wedge resection	No	DFD (2.4-y)
6	2008, Makhlouf	68	F	20	Monophasic	Wedge resection	No	AWOD at 2.4-y
7	2008, Makhlouf	29	M	28	Monophasic	Partial gastrectomy	No	AWOD at 18.7-y
8	2008, Makhlouf	54	F	30	Monophasic	Partial gastrectomy	No	N/A
9	2008, Makhlouf	58	F	30	Monophasic	Wedge resection	No	AWOD at 1.8-y
10	2008, Makhlouf	37	F	40	Monophasic	Partial gastrectomy	No	DFOC (4-y)
11	2008, Makhlouf	50	M	60	Monophasic	Tumor resection	Chemotherapy	AWD at 6-m
12	2008, Makhlouf	66	F	150	Monophasic	Total gastrectomy	No	N/A
13	2008, Makhlouf	42	M	80	Biphasic	Partial gastrectomy	Chemotherapy	DFD (2.1-y)
14	2012, Wang	38	F	75	Monophasic	Tumor resection	Chemotherapy	AWD at 6 months
15	2012, Sinniah	44	F	47	Monophasic	Wedge resection	No	AWOD at 3.3-y
16	2013, Sahara	22	M	25	Monophasic	Wedge resection	No	N/A
17	2013, Kamata	42	F	35	Monophasic	Partial gastrectomy	No	AWOD at 6-y
18	2014, Torres Rivas	44	M	150	Monophasic	Total gastrectomy	No	AWOD at 1.5-y
19	2014, Michot	62	M	38	Monophasic	Total gastrectomy	No	AWOD at 9-m
20	2015, Romeo	50	F	80	Monophasic	N/A	N/A	N/A
21	2015, Romeo	36	M	60	Poorly differentiated	N/A	N/A	AWD at 3-y
22	2015, Romeo	37	M	60	Monophasic	N/A	N/A	N/A
23	2015, Romeo	26	M	N/A	Monophasic	N/A	N/A	AWD at 15.4-y
24	2015, Romeo	58	M	100	Monophasic	N/A	N/A	DFD (6 months)
25	2015, Romeo	21	M	100	Monophasic	N/A	N/A	AWOD at 4-y
26	2015, Romeo	36	M	60	Biphasic	N/A	N/A	AWOD at 4-y
27	2015, Romeo	54	F	38	Monophasic	N/A	N/A	N/A
28	2015, Romeo	49	F	35	Monophasic	Tumor resection	No	N/A
29	2015, Romeo	35	F	120	Monophasic	Tumor resection	Chemotherapy	AWD at 4-y
30	2015, Wong	49	F	35	Monophasic	Tumor resection	No	AWOD at 10-m
31	2015, Wong	35	F	120	Monophasic	Tumor resection	Chemotherapy	AWD at 2-y
32	2017, So	51	F	17	Monophasic	Partial gastrectomy	No	AWOD at 2-m
33	2017, Hu	58	M	63	Monophasic	Wedge resection	N/A	AWD at 7-m
34	2018, Olsen	57	M	18	Monophasic	Wedge resection	No	AWOD
35	2018, Fuente	42	M	30	Monophasic	Tumor resection	No	AWOD at 1-y
36	2018, Ogino	27	F	20	N/A	Partial gastrectomy	N/A	AWOD at 6-m
37	2019, Bialik	26	M	80	Monophasic	Partial gastrectomy	Chemotherapy	AWOD
38	2020, Manohar	13	M	N/A	N/A	N/A	N/A	N/A
39	2020, Wong	54	M	16	Monophasic	Wedge resection	No	AWOD at 1.3-y
40	Current case	32	F	35	Biphasic	Wedge resection	No	AWOD at 8-m

Abbreviations: AWOD = Alive without disease; DFD = Died from disease; DFOC = Died from other causes; AWD = Alive with disease; N/A = Not available; m = month(s); y = year(s).

Best therapeutic approach for gastric synovial sarcoma is surgical resection with no specific excisional technique recommended. No invasion of regional lymph node areas has been reported from primary gastric SS, so lymph node dissection may reasonably be avoided. Benefit of adjuvant chemotherapy remains very uncertain as it is also questioned for all SS [19].

## 4 Conclusion

Gastric SS is a very uncommon neoplasia although it is henceforth a described entity. Immunohistochemical detection by FISH test of 18q11.2 translocation expression is needed to make the diagnosis of SS. Best therapeutic approach for these tumors remains surgical resection with no specific excisional technique recommended with regard to literature.

## Declaration of Competing Interest

The authors report no declarations of interest.

## Funding

This research did not receive any specific grant from funding agencies in the public, commercial, or not-for-profit sectors.

## Ethical approval

All investigators ensure that the conduct of this study is in accordance with the ethical standards of their respective institution as laid down in the 1964 Declaration of Helsinki.

## Consent

Written informed consent was obtained from the patient for publication of this case report and any accompanying images. A copy of the written consent is available for review by the Editor-in-Chief of this journal on request.

## Author contribution

CMC, FF, SBQ wrote the manuscript. KA performed the surgery. CG did the pathological analysis. All authors read and approved the final manuscript.

## Registration of research studies

Not applicable.

## Guarantor

Charles Marchand Crety, MD.

## Provenance and peer review

Not commissioned, externally peer-reviewed.
